# Extended-spectrum β-lactamase prevalence and virulence factor characterization of enterotoxigenic *Escherichia coli* responsible for acute diarrhea in Nepal from 2001 to 2016

**DOI:** 10.1186/s13756-018-0377-2

**Published:** 2018-07-20

**Authors:** Katie R. Margulieux, Apichai Srijan, Sirigade Ruekit, Panida Nobthai, Kamonporn Poramathikul, Prativa Pandey, Oralak Serichantalergs, Sanjaya K. Shrestha, Ladaporn Bodhidatta, Brett E. Swierczewski

**Affiliations:** 1 Department of Enteric Diseases, Armed Forces Research Institute of Medical Sciences, 315/6 Rajvithee Road, Bangkok, 10400 Thailand; 2 CIWEC Hospital and Travel Medicine Clinic, Kathmandu, Nepal; 3 Walter Reed/AFRIMS Research Unit Nepal, Kathmandu, Nepal; 4 Present Address: Bacterial Diseases Branch, Walter Reed Army Institute of Research, Silver Spring, MD USA

**Keywords:** ETEC, ESBL, Nepal

## Abstract

**Background:**

Multidrug-resistant (MDR) Gram-negative bacterial species are an increasingly dangerous public health threat, and are now endemic in many areas of South Asia. However, there are a lack of comprehensive data from many countries in this region determining historic and current MDR prevalence. Enterotoxigenic *Escherichia coli* (ETEC) is a leading cause of both acute infant diarrhea and traveler’s diarrhea in Nepal. The MDR prevalence and associated resistance mechanisms of ETEC isolates responsible for enteric infections in Nepal are largely unknown.

**Methods:**

A total of 265 ETEC isolates were obtained from acute diarrheal samples (263/265) or patient control samples (2/265) at traveler’s clinics or regional hospitals in Nepal from 2001 to 2016. Isolates were screened for antibiotic resistance, to include extended spectrum beta-lactamase (ESBL) production, via the Microscan Automated Microbiology System. ETEC virulence factors, specifically enterotoxins and colonization factors (CFs), were detected using multiplex PCR, and prevalence in the total isolate population was compared to ESBL-positive isolates. ESBL-positive isolates were assessed using multiplex PCR for genetic markers potentially responsible for observed resistance.

**Results:**

A total of 118/265 (44.5%) ETEC isolates demonstrated resistance to ≥2 antibiotics. ESBL-positive phenotypes were detected in 40/265 isolates, with isolates from 2008, 2013, 2014, and 2016 demonstrating ESBL prevalence rates of 1.5, 34.5, 31.2, and 35.0% respectively. No difference was observed in overall enterotoxin characterization between the total ETEC and ESBL-positive populations. The CFs CS2 (13.6%), CS3 (25.3%), CS6 (30.2%), and CS21 (62.6%) were the most prevalent in the total ETEC population. The ESBL-positive ETEC isolates exhibited a higher association trend with the CFs CS2 (37.5%), CS3 (35%), CS6 (42.5%), and CS21 (67.5%). The primary ESBL gene identified was *bla*_CTX-M-15_ (80%), followed by *bla*_SHV-12_ (20%) and *bla*_CTX-M-14_ (2.5%). The beta-lactamase genes *bla*_TEM-1_ (40%) and *bla*_CMY-2_ (2.5%) were also identified. It was determined that 42.5% of the ESBL-positive isolates carried multiple resistance genes.

**Conclusion:**

Over 30% of ETEC isolates collected post-2013 and evaluated in this study demonstrated ESBL resistance. Persistent surveillance and characterization of enteric ETEC isolates are vital for tracking the community presence of MDR bacterial species in order to recommend effective treatment strategies and help mitigate the spread of resistant pathogens.

## Background

Multidrug-resistant (MDR) Gram-negative bacterial species are an established prominent international public health threat [1]. MDR pathogens have an especially high prevalence in South Asia where bacterial infections are common and antibiotic use is widely unregulated [2–4]. A lack of comprehensive surveillance programs may result in the unchecked spread of MDR Gram-negative bacterial species and their associated resistance mechanisms. Developing countries with an established national surveillance network, such as Nepal, do not have the resources to perform in-depth isolate characterization to comprehensively identify resistance mechanisms carried by MDR bacterial isolates [5–7]. It is imperative that both archived and current bacterial isolates from underdeveloped regions undergo extensive MDR characterization to inform national strategies designed to halt the continuing spread of these dangerous pathogens.

Diarrheagenic *Escherichia coli* is a ubiquitous presence in the developing world and is responsible for a range of enteric infections [8–10]. Enterotoxigenic *E. coli* (ETEC) is defined by production of 1–3 enterotoxins, and is one of the leading causes of both acute infant diarrhea and traveler’s diarrhea worldwide, including Nepal [11–14]. ETEC attaches to and colonizes cells in the small intestine along the epithelial surface through the interaction of bacterial colonization factors (CFs) with the intestinal cell wall [15, 16]. After the establishment of intestinal colonization, the bacterial cells produce heat-labile enterotoxins (LT) and/or heat-stable enterotoxins (STh or STp) that target vital cellular processes [8, 17, 18]. The production of these enterotoxins most commonly results in abdominal cramping and watery diarrhea, with more severe cases also presenting with fever and nausea. ETEC infections are typically self-limiting with resolution of symptoms 3–7 days after initial onset and a recommended treatment strategy of rest and oral rehydration therapy [8, 19]. In spite of ongoing efforts to develop an ETEC vaccine, no viable vaccine candidates have been produced thus far [20, 21]. ETEC virulence factors, such as enterotoxins and CFs, are potential targets for vaccines or therapeutic candidates, and determination of regional prevalence rates and specific virulence factors may serve to inform the development of globally effective vaccines [15, 18–21].

Severe ETEC cases presenting with cholera-like watery diarrhea that do not resolve through oral rehydration therapy may require treatment with antibiotics [8, 10]. Initial treatment has historically been administration of first-line beta-lactams and quinolones. However, the evolution and global dissemination of multiple classes of antimicrobial resistance mechanisms has led to an increase of MDR ETEC infections that are insensitive to first-line antibiotics [11, 22]. Beta-lactamases and Extended-spectrum β-lactamases (ESBLs) confer resistance to a number of antibiotic classes, such as penicillins, extended-spectrum cephalosporins, and monobactams [23, 24]. Global dissemination of these mechanisms has occurred through clonal expansion and/or horizontal transfer of genetic resistance mechanisms between bacterial species [24]. Bacterial pathogens carrying ESBL resistance mechanisms are commonly associated with, and detected in, hospital-acquired infections. However, these antibiotic resistance mechanisms have spread to community-acquired isolates, and are increasingly identified in acute diarrhea and other enteric infections [12, 25–30]. ETEC infections are a large cause of acute diarrhea cases in Nepal, but prevalence rates of MDR ETEC isolates, both from a historic or current perspective, are unknown.

The current study determined antibiotic resistance and virulence factors of ETEC isolates previously identified from clinical acute diarrheal samples (263/265) or patient control samples (2/265) collected in Nepal between 2001 and 2016. The ETEC isolates were assayed to determine antimicrobial resistance profiles (including ESBL production) and virulence factor characterization, specifically enterotoxins and colonization factors. Antimicrobial resistance studies that encompass retrospective and current clinical isolates are vital to understanding the regional emergence of ESBL producing organisms, the existing treatment challenges, and the potential for ongoing dissemination. This study serves as a thorough investigation of antibiotic resistance in ETEC isolates from acute diarrheal samples in Nepal, and highlights the need for interventions that may address and stem the continual spread of MDR mechanisms in community settings.

## Methods

### Bacterial strains, media, and chemicals

A total of 265 ETEC isolates were previously isolated and identified from clinical acute diarrheal samples (263/265) or patient control samples (2/265) collected from hospitals and travel clinics in Nepal from 2001 to 2016. The isolates were archived for long-term storage at − 20 °C in Luria Bertani (LB) broth + 20% glycerol at the Armed Forces Research Institute of Medical Sciences (AFRIMS) in Bangkok, Thailand. The archived ETEC isolates were grown from frozen glycerol stocks on MacConkey agar (MAC) plates overnight at 37 °C. Isolated colonies were re-streaked on tryptic soy agar (TSA) plates, blood agar plates (BAP), or colonization factor agar (CFA) plates in preparation for further phenotypic analysis or DNA isolation.

### Antimicrobial susceptibility testing

All ETEC isolates were screened for ESBL production using the Microscan, Walkaway 40 automated system with Negative Breakpoint Combo 34 panels as per manufacturer’s instructions (Beckman Coulter, West Sacramento, CA, USA). ESBL-positive isolates were identified by automatic susceptibility testing panel results by assessing bacterial susceptibility to cefotaxime and ceftazidime with or without clavulanic acid. ESBL-positive ETEC isolates were confirmed through disk diffusion assay against beta-lactam combination agents using both cefotaxime (30 μg) and ceftazidime (30 μg) with or without clavulanic acid (10 μg) following Clinical and Laboratory Standards Institute guidelines [31]. A ≥ 5 mm increase in zone diameter for either antimicrobial agent tested in combination with clavulanate and the zone diameter of the agent when tested along indicated ESBL production.

### Multiplex PCR for ETEC virulence factors

The previously identified and archived ETEC isolates were re-tested to confirm the presence of ETEC enterotoxin genes (*lt*, *sth*, and *stp*) using multiplex PCR as previously described in detail [32]. ETEC colonization factors (CFA/1, CS1, CS2, CS3, CS4, CS5, CS6, CS7, CS8, CS12, CS13, CS14, CS15, CS17, CS18, CS19, CS20, CS21, and CS22) were identified using multiplex PCR as previously described in detail [32].

### Multiplex PCR for ESBL gene identification and DNA sequencing

ETEC isolates that tested positive for ESBL production were further characterized to determine potential genetic elements responsible for the observed antimicrobial resistance through multiplex PCR as described previously [33]. All PCR primers and conditions used for beta-lactamase or ESBL characterization were listed previously [33]. The genes identified from the multiplex PCR assays were sequenced to determine variant type. Genetic sequencing services were contracted through AITbiotech (Singapore) or First BASE (Selangor, Malaysia). The PCR products were prepared and sent for sequencing according to company instruction. The nucleotide sequences were analyzed with Sequencher software version 5.3 and BLAST software (http://www.Ncbi.nlm.nih.gov/BLAST).

### Statistics

Statistical analyses were performed using GraphPad QuickCalcs. Experimental groups were analyzed using a chi-square with Fisher’s exact test with a two-tailed *p* value of ≤0.05 considered statistically significant.

## Results

### Antibiotic resistance characterization of ETEC isolates

A total of 117/265 (44.1%) ETEC isolates demonstrated full susceptibility to all antibiotics tested, with an additional 30/265 (11.3%) resistant to only a single antibiotic tested. The remaining 118/265 (44.5%) isolates demonstrated resistance to ≥2 antibiotics tested (Table 1). Antibiotic resistance to ampicillin was most common (113/265, 42.6%), followed by trimethoprim/sulfamethoxazole (77/265, 29.1%), tetracycline (74/265, 27.9%), and ampicillin/ sulbactam (32/265, 12.1%). A lesser number of isolates were shown to be resistant to ciprofloxacin (15/265, 5.7%) and no isolates showed antibiotic resistance to ertapenem. Of the 118 isolates with increased drug-resistance, 40 demonstrated phenotypic production of ESBLs with resistance to the extended-spectrum β-lactams cefotaxime (40/40, 100%), ceftazidime (39/40, 97.5%), and/or ceftriaxone (35/40, 87.5%) (Table 1). ESBL-production in all positive ETEC isolates was confirmed through a disk diffusion test. A single isolate from 2008 was positive for ESBL production (1/62, 1.5%). The remaining ESBL-positive ETEC isolates were collected during 2013 (8/21), 2014 (10/32), and 2016 (21/60), resulting in yearly prevalence rates of 34.5, 31.2, and 35.0%, respectively (Table 1). Of the ESBL-positive ETEC isolates, the majority were recovered from acute diarrheal samples (39/40, 97.5%), however, 1/40 (2.5%) was recovered from a patient control sample.

**Table 1:** Total number of ETEC isolates, isolates resistant to ≥2 antibiotics, and isolates that display ESBL-positive phenotypes per year

Year	Total # of Isolates	Isolates *R* ≥ 2 Antibiotics (%)	ESBL Positive Isolates (%)
2001	12	9 (75)	0
2002	30	5 (16.7)	0
2003	5	3 (60)	0
2007	34	8 (23.5)	0
2008	62	25 (40.3)	1 (1.5)
2009	8	4 (50)	0
2012	1	0	0
2013	21	11 (52.4)	8 (35.6)
2014	32	14 (43.8)	10 (31.2)
2016	60	39 (65)	21 (35)
Total	265	118 (44.5)	40 (15.1)

### ETEC enterotoxin gene characterization and prevalence

Detection of the ETEC enterotoxin genes *lt*, *sth*, and/or *stp* was performed for all isolates. The enterotoxin gene *lt* was detected in a total of 132/265 (49.8%) isolates, *sth* was detected in a total of 152/265 (57.3%) isolates, and *stp* was detected in a total of 72/265 (27.5%) isolates. Multiple enterotoxin genes were detected in 88/265 (33.2%) isolates, and enterotoxin gene combinations prevalence are described in Table 2. The enterotoxin combination of *sth* only was significantly detected less frequently in ESBL-positive isolates compared to ESBL-negative isolates (15% vs. 30.9%; *p* = 0.0156). No other enterotoxin gene (by total percent detected or by combinations) demonstrated a statistically significant difference in prevalence rates between the two populations.

**Table 2:** Enterotoxin gene detection within total ETEC population and ESBL-positive population

Enterotoxin(s)	Total *n* = 265 (%)	ESBL Positive *n* = 40 (%)
*lt*	132 (49.8)	23 (57.5)
*sth*	152 (57.3)	21 (52.5)
*stp*	73 (27.5)	13 (32.5)
Multiple	88 (33.2)	17 (42.5)
Combinations		
*lt* only	44 (16.6)	6 (15)
*sth* only	82 (30.9)	6 (15)
*stp* only	51 (19.2)	11 (27.5)
*lt*/*sth*	66 (24.9)	15 (37.5)
*lt*/*stp*	18 (6.8)	2 (5)
*lt/sth/stp*	4 (1.5)	0

### ETEC CF characterization and prevalence

CFs were detected in 222/265 (83.8%) of the total ETEC isolates, and multiple CFs were detected in 162/265 (61.1%) isolates. CS2 (13.6%), CS3 (25.3%), CS6 (30.2%), and CS21 (62.6%) were the most prevalent CFs identified in the total ETEC population. The ESBL-positive ETEC isolates showed a trend of higher association with CS2 (37.5%), CS3 (35%), CS6 (42.5%), and CS21 (67.5%) compared to the total population. The ESBL-positive ETEC population had a statistically significant higher prevalence rate compared to the ESBL-negative population for CS2 (37.5.0% vs. 9.3%; *p* = 0.0002). Conversely, the ESBL-negative population had a statistically significant higher prevalence of CS1 compared to the ESBL-positive population (10.2% vs 0.0%; *p* = 0.0311). The multiple CF combinations CS6/CS21 and CS2/CS3/CS21 were the most prevalent in both the total population and ESBL-positive population (Table 3). The CF combination CS2/CS3/CS21 was statistically significantly higher in the ESBL-positive ETEC population when compared to the ESBL-negative ETEC population (25.0% vs. 7.1%; *p* = 0.0018). No other CFs or CF combinations were statistically significant between the two populations, despite overall higher prevalence trends demonstrated in the ESBL-positive ETEC population for many CFs and CF combinations.

**Table 3:** Colonization factor characterization within total ETEC population and ESBL-positive ETEC population

Colonization Factor	Total *n* = 265 (%)	ESBL Positive *n* = 40 (%)
CFA/1	18 (6.8)	3 (7.5)
CS1	23 (8.7)	0
CS2	36 (13.6)	15 (37.5)
CS3	67 (25.3)	14 (35)
CS4	8 (3)	0
CS6	80 (30.2)	17 (42.5)
CS7	5 (1.9)	0
CS8	11 (4.2)	0
CS12	6 (2.3)	2 (5)
CS13	1 (0.4)	0
CS14	5 (1.9)	0
CS17	10 (3.8)	0
CS20	5 (1.9)	0
CS21	166 (62.6)	27 (67.5)
None Detected	43 (16.2)	3 (7.5)
Multiple CFs	162 (61.1)	30 (75)
Combinations		
CS6/CS21	55 (20.8)	12 (30)
CS2/CS3/CS21	26 (9.8)	10 (25)
CS1/CS3/CS21	22 (8.3)	0

### ESBL gene identification

ESBL-positive ETEC isolates were screened for carriage of known prevalent ESBL and/or beta-lactamase genes using a multiplex PCR assay [33]. The most prevalent ESBL gene detected was *bla*_CTX-M_ group 1 with 32/40 (80%) of isolates harboring this gene. This gene was further identified as *bla*_CTX-M-15_ through PCR sequencing analysis (Table 4). Additionally, the ESBL genes *bla*_SHV-12_ and *bla*_CTX-M-14_ were identified with prevalence rates of 20% (8/40) and 2.5% (1/40), respectively. The beta-lactamase genes *bla*_TEM-1_ (16/40, 40%) and *bla*_CMY-2_ (1/40, 2.5%) were also shown to be carried in the ESBL-positive ETEC population. A total of 17/40 (42.5%) isolates were shown to carry multiple ESBL and/or beta-lactamase genes (Table 4). Notably, the multiplex PCR screen did not identify any ESBL or beta-lactamase genes in one phenotypically ESBL-positive ETEC isolate. The multiplex PCR assay performed in this study screens for prevalent resistance genes, but is not a comprehensive investigation of ESBL genes. As such, additional analysis of this isolate may identify the presence of less prevalent or novel ESBL genes.

**Table 4:** Determination of ESBL and beta-lactamase gene type

		No. of Isolates with Gene(s) Detected (%)						
Year	No. of Isolates	*bla*_CTX-M-15_	*bla*_CTX-M-14_	*bla*_SHV-12_	*bla*_TEM-1_	*bla*_CMY-2_	No Genes Detected	Multiple Genes
2008	1	1 (100)	0	0	0	0	0	0
2013	8	8 (100)	0	1 (12.5)	6(75)	0	0	7 (87.5)
2014	10	8 (80)	1 (10)	1 (10)	4(40)	0	0	4 (40)
2016	21	15 (71.4)	0	6 (28.6)	6(28.6)	1 (4.8)	1 (4.8)	6 (23.8)
Total	40	32 (80)	1 (2.5)	8 (20)	15(37.5)	1 (2.5)	1 (2.5)	17 (42.5)

## Discussion

MDR Gram-negative bacterial pathogens are a global public health threat [1, 6]. ESBL-producing bacterial strains were first detected in, and were mostly limited to, hospital-associated infections, but began being detected in community-associated infections beginning in the early 2000’s [25, 27, 28, 34]. Initial reports of community-associated ESBL-producing *E. coli* strains, including reports originating from Nepal, are mainly comprised of pathogens isolated from urinary tract or blood stream infections [35–39]. However, community-associated infections isolated from enteric MDR pathogens are being increasingly reported in South and Southeast Asia [8, 11, 12, 22, 26, 30].

ETEC is an enteric pathogen that results in both acute infant diarrhea and traveler’s diarrhea [8, 10, 15]. ESBL-producing ETEC may result in treatment failures for infections that were previously easily treatable with first-line antibiotic administration. In the current study, only a single ESBL-positive ETEC isolate was identified from isolates archived in 2001–2009 from Nepal. However, over 30% of the ETEC isolates collected after 2013 phenotypically display ESBL production. This suggests that ESBL resistance mechanisms in Nepal have expanded relatively recently into community-associated bacterial pathogens, and represents a new threat to both the community as well as international travelers in the region. Notably, international travelers have been shown to be a potential vector for the spread of resistant bacterial pathogens through acquisition during travel, contributing to the global dissemination of MDR enteric pathogens [40]. The combination of established community prevalence and additional spread by infected travelers may contribute to local and global dissemination of ESBL-positive ETEC isolates.

ETEC virulence factors, such as enterotoxins and CFs, are of particular interest as potential targets for infection intervention as vaccine or therapeutic targets [15, 18, 21]. The expression of enterotoxins and CFs are essential for ETEC colonization of the small intestine and resulting pathogenesis. Blocking bacterial cell adhesion to the small intestine and/or neutralizing enterotoxins results in a limited ETEC infection and significantly reduces infection morbidity [15, 18, 21]. The currently reported prevalence of the enterotoxins LT, STh, and STp in this study were comparative to previous studies reviewed and analyzed by Isidean, et al. [18]. The most prevalent CFs in the current study were CS21, CS6, CS3, and CS2, while the most prevalent CFs in previously conducted studies reviewed by Isidean et al were CFA/1, CS6, and CS21 [18]. Notably, CFA/1 was detected at a lower rate in the current study than previously reported (6.8 vs 20%), indicating CFA/1 may not be a priority target for ETEC interventions in South Asia [18]. Overall, the current study suggests vaccines and therapeutics targeting the enterotoxins LT and STh, and the CF’s CS21, CS6, CS2, and CS3 would likely be most efficacious against ETEC infections in South Asia. This study demonstrated that these virulence factors are also prevalent in the ESBL-positive ETEC population, a group that is important to target for vaccine efficacy as MDR ETEC continues to emerge and spread throughout the region. It is important that ongoing studies characterizing virulence factors in MDR ETEC populations are performed to determine relevant intervention strategies against currently circulating strains.

Many ESBL genes have been reported as actively circulating in South Asia in hospital- and community-associated infections, including *bla*_CTX-M_ group 1 and group 9, *bla*_PER_, *bla*_VEB_, *bla*_GES_, and *bla*_SHV_ [41]. Within the gene family *bla*_CTX-M,_ *bla*_CTX-M-15_ and *bla*_CTX-M-14_ are the most common type of ESBL found worldwide [27, 42]. Aligning with the global findings of gene prevalence, previous studies conducted in Nepal have identified *bla*_CTX-M-15_ as prevalent in hospital-associated *E. coli* infections, while *bla*_TEM_ and *bla*_SHV_ were also detected at a lower frequencies [43, 44]. In the current study, *bla*_CTX-M-15_ was detected in the majority of ESBL-positive ETEC isolates. The genes *bla*_SHV-12_, *bla*_CTX-M-14_, *bla*_TEM-1_, and *bla*_CMY-2_ were also identified at lower rates, and multiple resistance genes were detected in 40% of the characterized isolates. Importantly, ESBL resistance mechanisms are often located on mobile plasmids [42, 45, 46]. As such, enteric ETEC strains that are ESBL-positive may potentially serve as a community reservoir in Nepal for the dissemination of ESBL resistance mechanisms via clonal expansion and/or horizontal transfer to other bacterial species that do not result in enteric infections. A large percentage of ETEC isolates identified in the current study contain multiple resistance mechanisms, suggesting that horizontal transfer of ESBL genes is actively occurring within enteric bacterial pathogens. Interestingly, this study identified an ESBL-positive ETEC isolate from a patient control sample, absent of diarrheal symptoms, indicating the established presence of asymptomatic MDR pathogens in the community that may contribute to gene dissemination. Additional studies are necessary to observe the source and spread of ESBL resistance mechanisms in community-associated infections in Nepal.

Limitations of the current study include small sample sizes per year and the lack of clinical patient information. These limitations serve to highlight the need for additional long-term, comprehensive enteric pathogen surveillance to track ongoing MDR emergence and spread in community settings. ESBL-positive *E. coli* often have limited treatment options, such as carbapenem antibiotics. However, the efficacy of these treatment options are compromised by increased prevalence of carbapenemase resistance mechanisms, such as *bla*_NDM-1_, *bla*_KPC_, and *bla*_IMP_. No carbapenemase-producing ETEC were identified in the current study, but continued surveillance is imperative for tracking if, or more likely when, community-associated MDR ETEC isolates acquire carbapenem-resistance mechanisms in Nepal.

## Conclusions

MDR Gram-negative bacterial species pose a dangerous threat to global public health, including community-associated MDR enteric infections that are increasingly prevalent. The current study demonstrated that ESBL resistance mechanisms have spread to community-associated pathogens in Nepal, with over 30% of ETEC isolates collected after 2013 demonstrating ESBL production. Both retrospective and recent surveillance studies of ETEC isolates identified from acute diarrheal clinical samples are vital for tracking the community presence of emerging antibiotic resistance mechanisms. Consistent, long-term surveillance may lead to better informed treatment options, alternative therapeutic development, and community-based interventions to mitigate the spread of resistant bacterial strains beyond hospital environments.

## Abbreviations

AFRIMS: Armed Forces Research Institute of Medical Sciences
CFs: Colonization factors
ESBL: Extended spectrum beta-lactamase
ETEC: Enterotoxigenic *Escherichia coli*
LT: Heat-labile enterotoxin
MDR: Multidrug resistance
ST: Heat-stabile enterotoxin
